# The Planning of Hospitals

**Published:** 1895-02-09

**Authors:** 


### THE PLANNING OF HOSPITALS.

It is time that some practical steps were taken by the County Councils, and especially by the London County Council and the Metropolitan Asylums Board, when plans for new asylums are put up for competition, to ensure that the best plans shall be selected. Heretofore it has been customary to apply to the Royal Institute of British Architects, asking them to nominate an assessor. The plan pursued by the Institute is, we understand, to select the names of three or four of their own senior fellows, quite irrespective of their knowledge of hospital and asylum construction, and to send them to whoever may write for a list. There can be no doubt that the public interest demands that the County Councils and others who have to erect large buildings of this description should set themselves to work to ascertain who is the best expert, that is, who knows enough of the subject all round, to secure that the best plan on its merits shall be chosen. If this plan were pursued, quite irrespective of any particular competition by all local authorities throughout the country, they might save considerable sums of money and avoid many evils which have resulted from the present unsatisfactory method of selecting an assessor.

